# Long non-coding MELTF Antisense RNA 1 promotes and prognosis the progression of non-small cell lung cancer by targeting miR-1299

**DOI:** 10.1080/21655979.2022.2063563

**Published:** 2022-04-20

**Authors:** Jin Chai, Li Qin, Guangxin Zhang, Peiyan Hua, Chengyan Jin

**Affiliations:** a Department of Pharmacy, The Second Hospital of Jilin University, Changchun, Jilin, China; b Department of Thoracic Surgery, The Second Hospital of Jilin University, Changchun, Jilin, China

**Keywords:** NSCLC, lncRNA MELTF-AS1, miR-1299, prognosis, biomarker

## Abstract

This paper explored the influence of long non-coding MELTF Antisense RNA 1 (lncRNA MELTF-AS1) on the prognosis of non-small cell lung cancer (NSCLC), and further deepened the understanding of NSCLC. A total of 130 patients with NSCLC participated in current study to detect and compare lncRNA MELTF-AS1 expression in cancer and normal tissues. Kaplan-Meier analysis and log-rank test were chosen to analyze the effect of MELTF-AS1 expression on the survival of patients within 5 years. The correlation between the expression of MELTF-AS1 and the clinical characteristics of NSCLC patients was analyzed, and the prognostic factors of NSCLC were analyzed by multivariate Cox regression. Subsequently, MELTF-AS1 expression in NSCLC cells were detected. The Cell Counting Kit-8 (CCK-8) and Transwell methods were selected to study the proliferation, migration capability and invasion level of NSCLC cells that silencing MELTF-AS1. Through the luciferase activity assay to explore the relationship between MELTF-AS1 and miR-1299, to further understand the effect of silencing MELTF-AS1 on NSCLC. MELTF-AS1 was increased in NSCLC tissues and cells. Silencing MELTF-AS1 suppressed the proliferation ability, migration capability and invasion level of NSCLC cells, which means that low expression of MELTF-AS1 may be more conducive to patient survival. In addition, through luciferase activity analysis and bioinformatics analysis, MELTF-AS1 has a negative effect on miR-1299, and silencing MELTF-AS1 enhanced miR-1299 expression in NSCLC cells. MELTF-AS1 is highly likely to be a promising prognostic biomarker, and associated with the progression of NSCLC.

## Graphical Abstract

**Figure:**
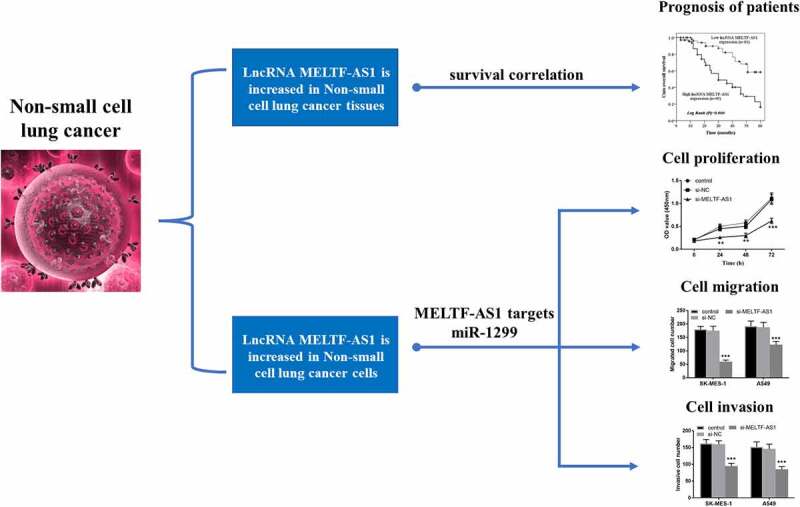

## Highlights

- TRB3 interacts with GRB2 in HG-triggered retinal pigment epithelial cells s
- TRB3 modulates inflammation and oxidative stress in DR through binding to GRB2
- TRB3 modulates cell damage in DR through binding to GRB2

## Introduction

The latest research shows that deaths from lung cancer accounting for about 18% of all cancer deaths [1]. About 85% of the histological subtypes of lung cancer are collectively referred to as non-small cell lung cancer (NSCLC), of which lung adenocarcinoma and lung squamous cell carcinoma are the most common subtypes [2–4]. The incidence of NSCLC currently ranks second in the world, and it is known that the incidence of men is higher than that of women [5]. Long-term smoking has been identified as the main cause of lung cancer, including NSCLC. Carcinogens in cigarettes can accelerate cell canceration [6,7]. In addition, environmental and genetic factors are influencing factors for NSCLC [8,9]. Except to surgery, chemotherapy and targeted drug therapy, methods such as drug combination therapy, molecular genetic testing and diagnosis have also been gradually applied [10]. However, the cure rate and survival rate of NSCLC are still very low, and the recurrence rate is high.

In recent years, research on long non-coding RNA (lncRNA) has continued to increase. It is understood that lncRNA is more than 200 nucleotides in length and cannot encode proteins, but it can regulate gene expression and malignant tumors [11–13]. It has been reported that the lncRNA MCM3AP-AS1 promoted the progression of NSCLC and might serve as a promising therapeutic target for NSCLC [14]. Recent studies have shown that lncRNA LINRIS knockdown might inhibit the proliferation of NSCLC cells by inhibiting miR-10a maturation [15]. Jiang and his colleagues found that lncRNA MELTF Antisense RNA 1 (lncRNA MELTF-AS1) has potential prognostic value for patients with renal cell carcinoma, providing new insights for tumor treatment [16]. However, lncRNA MELTF-AS1 has no specific mechanism and clinical relevance in NSCLC.

Therefore, based on the increasing number of NSCLC patients and the high recurrence rate within 5 years, there is an urgent need to discuss the molecular mechanism of NSCLC and monitor potential prognostic biomarkers to improve patient survival.

## Materials and methods

### Research object samples

A total of 130 Chinese NSCLC patients participated, and all patients did not receive any chemotherapy or other related tumor treatments before the study. NSCLC and normal tissues were removed, frozen with liquid nitrogen, and stored in a refrigerator [17]. The research was conducted in February 2014 and ended in June 2017. With the approval of ethics Committee ([2014] No. 041) and under the supervision of professional pathologists, the study was carried out in accordance with regulations, and all participants signed written informed consent. In the 5 years after the study, all participants were followed up through telephone follow-up, face-to-face interviews and other methods, which achieved the purpose of timely obtaining NSCLC patient information. Table 1 illustrated the correlation between the expression of MELTF-AS1 and clinical characteristics such as age, gender, tumor size, smoking status, and TNM staging.

**Table 1.** Correlation of the lncRNA MELTF-AS1 expression with clinical characteristics in NSCLC

Parameters	Patients (n = 130)	lncRNA MELTF-AS1 expression		*P*
		Low (n = 61)	High (n = 69)	
Age				0.991
≤ 60	64	30	34	
> 60	66	31	35	
Gender				0.886
Male	78	37	41	
Female	52	24	28	
Tumor size				0.081
≤ 5 cm	64	35	29	
> 5 cm	66	26	40	
Smoking status				0.701
Nonsmoker	62	28	34	
Smoker	68	33	35	
Differentiation				0.129
Well, Moderate	74	39	35	
Poor	56	22	34	
Lymph node metastasis				0.006
Negative	82	46	36	
Positive	48	15	33	
TNM stage				0.003
I, II	90	50	40	
III, IV	40	11	29	

Annotation: NSCLC, non-small cell lung cancer.

### Cultivation of NSCLC cells

Shanghai Cell Bank of the Chinese Academy of Sciences (Shanghai, China) provided four NSCLC cell lines (A549 and NCI-H650 were lung adenocarcinoma cell lines; HCC95 and SK-MES-1 were lung squamous cell carcinoma cell lines) and control cell lines (BEAS-2B). The cells involved in the experiment were cultured in Dulbecco’s modified Eagle medium (DMEM; GE Healthcare), supplemented with 10% fetal bovine serum (FBS; GE Healthcare) and 100 U/mL penicillin/streptomycin (Hyclone; GE Healthcare). The cells were washed with phosphate buffered saline (PBS; GE Healthcare) solution and placed in a 37°C humidified incubator containing 5% CO_2_ [18].

GenePharma Co. Ltd. (Shanghai, China) chemically synthesized miR-1299 mimic, mimic NC, miR-1299 inhibitor, and inhibitor NC. The cells were subcultured in 6-well plates. Then, Lipofectamine 2000 (Invitrogen; USA) was used to transfect RNA into cells.

### RT-qPCR

TRIZOL reagent (Thermo Fisher Scientific, Inc.) was selected to obtain total RNA in tissues and cells, and the purity and concentration of total RNA were determined via a spectrophotometer (Eppendorf) [19]. According to the requirements of the instructions, the Reverse Transcription Kit (Promega Corporation) was used to reverse transcribe RNA into cDNA. Then the SYBR®GREEN qPCR Super Mix Kit (Vazyme) was used to configure the reaction system, and the RT-qPCR assay was performed through the 7500 sequences detection system (Thermo Fisher Scientific, Inc.). The reaction system was 10 µL, including 2.5 µL cDNA, 5 µL 2× SYBR GREEN qPCR Super Mix, 0.5 µL primers and 2 µL ddH_2_O. Subsequently, GAPDH and U6 were used as endogenous controls, and the data was calculated and processed by 2^−ΔΔCt^ method. The primer sequences are as follows: MELTF-AS1 forward, 5´-GCGTTCACACTCATTACCC-3´ and reverse, 5´-CTATTCAGACCCCTTCACCC-3´ GAPDH forward, 5´-GAGCCACATCGCTCAGACAC-3´ and reverse, 5´-GCCCAATACGACCAAATCC-3´ U6 forward, 5´-GCTTCGGCAGCACATATACTAAAAT-3´ and reverse, 5´-CGCTTCACGAATTTGCGTGTCAT-3´.

### Cell proliferation ability, migration capability and invasion level assay

The cell proliferation ability of SK-MES-1 and A549 cells were measured according to the Cell Counting Kit-8 (CCK-8; Beyotime Institute of Biotechnology). The transfected cells were cultured in a 96-well culture plate (1 × 10^4^ cells/well), and 100 μL of DMEM medium containing 10% FBS was added to each well. Then added CCK-8 solution (10 µL), incubated in a humidified incubator containing 5% CO_2_ at 37°C for 4 hours, and the absorbance at 450 nm in each well was measured with a microplate reader (Thermo Fisher Scientific, Inc.).

The level of cell migration and invasion was detected by the Transwell (BD Biosciences, USA) method. One hundred mocroliter DMEM medium was placed on the upper chamber of Transwell, and DMEM medium containing 10% FBS was served into the lower chamber to induce cell migration. After incubation for 48 h, fixation with 4% paraformaldehyde for 20 min at room temperature. Then washing with PBS solution and staining with 0.1% crystal violet for 10 min. Cell invasion and migration levels were detected in similar steps, but Matrigel (BD Biosciences, USA) was applied to the upper Transwell chamber in advance. Finally, NSCLC cells were cleaned by PBS solution again, and an inverted microscope (Olympus Corporation) was used for random image sampling. Each assay was repeated for 3 times.

### Luciferase activity detection assay

The luciferase reporter gene detection system (Promega Corporation) was selected to measure A549 cells. The wild-type and mutant-type lncRNA MELTF-AS1 were cloned into pmirGLO vector (Promega Corporation), and WT-MELTF-AS1 and MUT-MELTF-AS1 were constructed respectively. Subsequently, A549 cells were co-transfected with miR-1299 mimic, miR-1299 inhibitor, mimic NC or inhibitor NC and WT-MELTF-AS1 or MUT-MELTF-AS1. After transfection for 48 hours and washing with PBS solution, the luciferase activity was measured with a dual-luciferase reporter gene detection system. Argonaute 2 (Ago2) is a key component of RNA-induced silencing complex (RISC), and miRNAs regulate the expression of their target genes by binding to Ago2 [20]. In this study, anti-Ago2 RNA immunoprecipitation (RIP) assay was performed. The RNA transcript was combined with Ago2 antibody, and IgG was used as a negative control. Each assay was repeated for 3 times.

### Statistical analysis

Data processing and statistical analysis were performed by SPSS 20.0 software and GraphPad Prism 5.0 software (USA). The measurement data were expressed as mean ± standard deviation (x ± s), and count data were expressed by the χ^2^ test. The prognostic significance of MELTF-AS1 was detected by Kaplan-Meier method. Multivariate Cox regression method was chosen to analyze the prognostic factors of NSCLC patients. Each assay was repeated for 3 times. *P* < 0.05 represents statistical significance.

### Results

The prognostic and therapeutic potential of lncRNA MELTF-AS1 in NSCLC is the main thrust of this study. To understand the possibility of MELTF-AS1 as a prognostic biomarker, RT-qPCR was used to detect the expression level of MELTF-AS1 in tissues and cells. The effects of silencing MELTF-AS1 on the proliferation, migration and invasion of NSCLC cells were discussed by methods such as CCK-8 and Transwell. The targeting effect of MELTF-AS1 on miR-1299 by luciferase activity detection assay.

### Expression of MELTF-AS1 increased

**Figure 1.**
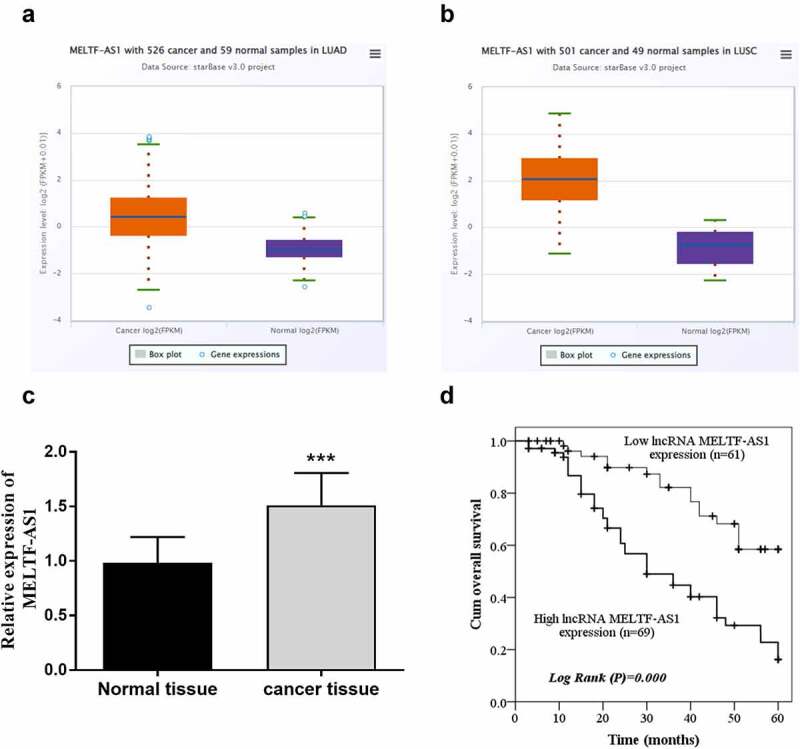
MELTF-AS1 in tissues and the overall survival of different expressions of MELTF-AS1 were detected by Kaplan-Meier approach. (a) MELTF-AS1 with 526 cancer and 59 normal samples in LUAD. (b) MELTF-AS1 with 501 cancer and 49 normal samples in LUSC. (c) The expression of MELTF-AS1 is increased in cancer tissues by RT-qPCR. (d) The overall survival of low-expression MELTF-AS1 was significantly higher than that of high-expression MELTF-AS1 (log-rank *P* = 0.000). ***P < 0.001.

RT-qPCR was chosen to test and compare MELTF-AS1 expression in NSCLC and normal tissues, and Kaplan-Meier analysis and log-rank test were selected to analyze the effects of different expressions of MELTF-AS1 on overall survival. For the TCGA NSCLC cohort data, LUAD samples and LUSC samples were analyzed separately. The results are shown in Figure 1(a,b), MELTF-AS1 in NSCLC samples was higher than that in normal samples. Figure 1(c) exhibits that MELTF-AS1 was up-regulated about 1.5 times in cancer tissues. According to the average expression level of MELTF-AS1, it was consisted of high lncRNA MELTF-AS1 expression (n = 69) and low lncRNA MELTF-AS1 expression (n = 61). Figure 1(d) shows that the cum overall survival rate of low lncRNA MELTF-AS1 expression was higher than that of high lncRNA MELTF-AS1 expression within 5 years (log-rank *P* = 0.000).

### The possibility of lncRNA MELTF-AS1 as the prognosis of NSCLC

The correlation between MELTF-AS1 expression and clinical characteristics of Chinese NSCLC patients was studied, and the prognostic risk factors of NSCLC were analyzed by multivariate Cox regression. Table 1 reveals that lncRNA MELTF-AS1 expression was associated with lymph node metastasis (*P* = 0.006) and TNM stage (*P* = 0.003). There was no significant correlation with patient age, gender, tumor size, smoking status and differentiation. Table 2 shows that lncRNA MELTF-AS1 (*P* < 0.001), lymph node metastasis (*P* = 0.013) and TNM stage (*P* = 0.007) were all related prognostic risk factors of NSCLC. These results implied that MELTF-AS1 has the potential as a prognostic biomarker of NSCLC. *P* < 0.05 represents statistical significance.

**Table 2.** Multivariate Cox analysis of clinical characteristics in relation to overall survival

	Multivariate analysis		
Characteristics	HR	95% CI	*P*
lncRNA MELTF-AS1	3.423	1.790–6.548	<0.001
Age	1.031	0.586–1.812	0.916
Gender	1.010	0.582–1.753	0.972
Tumor size	1.435	0.820–2.513	0.206
Smoking status	1.141	0.653–1.993	0.643
Differentiation	1.278	0.729–2.240	0.392
Lymph node metastasis	2.250	1.183–4.279	0.013
TNM stage	2.191	1.235–3.888	0.007

### Silencing MELTF-AS1 expression

**Figure 2.**
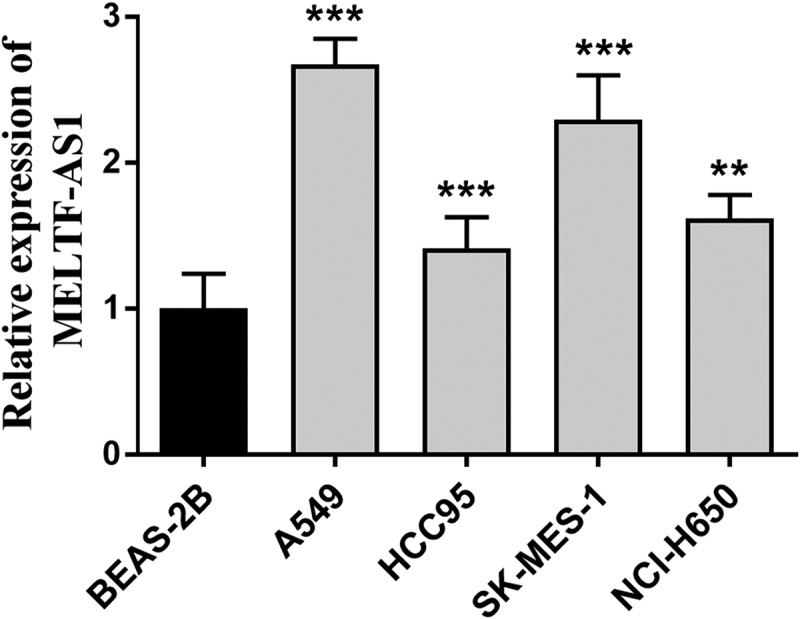
The expression of MELTF-AS1 in cancer cells and control cells was detected by RT-qPCR. MELTF-AS1 has increased expression in four cancer cells (A549, HCC95, SK-MES-1 and NCI-H650). **P < 0.01, ***P < 0.001.

RT-qPCR was used to detect and compare the relative expression of MELTF-AS1 in different NSCLC cells and normal cells, and to discuss the effects of silencing MELTF-AS1 on the proliferation capacity, migration and invasion levels of NSCLC cells. The relative expression of MELTF-AS1 in A549, HCC95, SK-MES-1 and NCI-H650 cells was determined, and the content of MELTF-AS1 increased compared with the control BEAS-2B, as shown in Figure 2. The most significant up-regulated A549 and SK-MES-1 cell lines were selected for subsequent assays.

**Figure 3.**
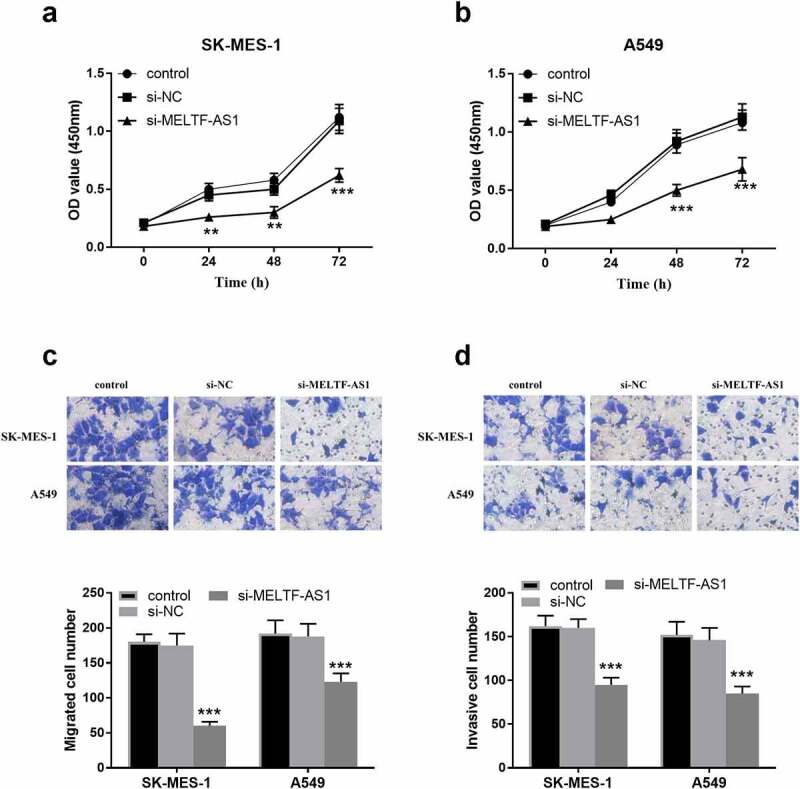
The effect of silencing MELTF-AS1 on the proliferation, migration capability and invasion levels of SK-MES-1 and A549 cells. (a) and (b) Proliferative capacity of SK-MES-1 and A549 cells were measured. (c) Migratory capability was measured by transwell assay. (d) Invasive level of SK-MES-1 and A549 cells were measured. **P < 0.01, ***P < 0.001.

In Figure 3(a,b), OD values at 450 nm in 0, 24, 48 and 72 h were detected respectively, indicating that the OD values of silencing MELTF-AS1 (si-MELTF-AS1) were lower than those of control and si-NC groups in SK-MES-1 and A549 cells. It was suggested that silencing MELTF-AS1 may down-regulate the proliferation of NSCLC cells. As displayed in Figure 3(c,d), si-MELTF-AS1 significantly reduced the number of migration and invasion cells in SK-MES-1 and A549 cells compared with the control group and si-NC groups, proved that silencing MELTF-AS1 inhibited the migration and invasion levels of cells.

### Luciferase activity assay and MELTF-AS1 interacted with miR-1299

**Figure 4.**
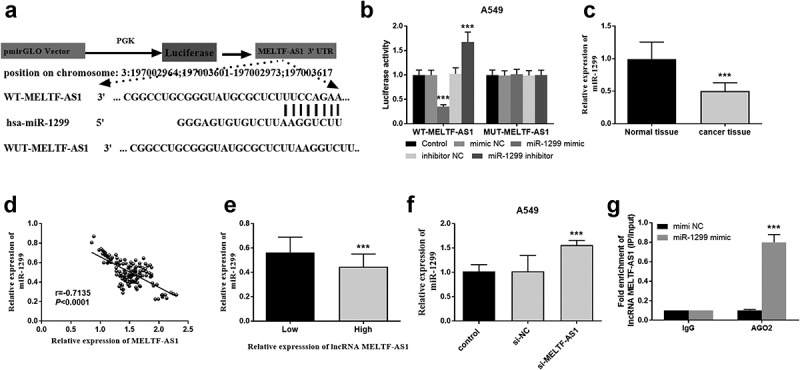
Luciferase activity detection and the correlation between MELTF-AS1 and miR-1299. (a) The binding sites of WT-MELTF-AS1 and miR-1299 at the 3'UTR of MELTF-AS1. (b) Luciferase activity was examined in A549 cells cotransfected with WT-MELTF-AS1 or MUT-MELTF-AS1 and miR-1299 mimic, miR-1299 inhibitor, mimic NC or inhibitor NC. (c) miR-1299 expression in cancer tissues was markedly reduced. (d) MELTF-AS1 and miR-1299 were negatively correlated (r = −0.7135, *P* < 0.0001). (e) High lncRNA MELTF-AS1 expression in NSCLC tissues reduced miR-1299. (f) In A549 cells, the expression of miR-1299 in silent MELTF-AS1 increased. (g) MiR-1299 mimic in anti-Ago2 antibody significantly increased MELTF-AS1. ***P < 0.001.

Luciferase activity was further explored in A549 cells and bioinformatics analysis was performed to discuss the interaction between MELTF-AS1 and miR-1299. Figure 4(a) shows that miR-1299 and MELTF-AS1 3'UTR form multiple base pairs. As shown in Figure 4(b), the luciferase activity of transfected WT-MELTF-AS1 cells was inhibited with the miR-1299 increased, while miR-1299 expression decreased, the luciferase activity increased. However, MUT-MELTF-AS1 transfected cells did not significantly affect luciferase activity. The expression level of miR-1299 down-regulated in cancer tissues in Figure 4(c). The relative expression of MELTF-AS1 and miR-1299 was negatively correlated. That is, the increased in the expression of MELTF-AS1 affect the expression of miR-1299 (r = −0.7135, *P* < 0.0001, Figure 4(d)). Figure 4(e) suggests that high lncRNA MELTF-AS1 expression decreased miR-1299 in NSCLC tissues compared with low lncRNA MELTF-AS1 expression. In A549 cells si-MELTF-AS1 increased the expression of miR-1299 as shown in Figure 4(f). It can be speculated that MELTF-AS1 expression may down-regulate miR-1299. The results of the anti-Ago2 RIP assay showed that miR-1299 mimic in anti-Ago2 antibody significantly increased MELTF-AS1, that is, MELTF-AS1 could bind to miR-1299 in NSCLC (Figure 4(g)). False discovery rate (FDR) correction used to identify the association between lncRNA MELTF-AS1 and miR-1299 using bioinformatics was applied to the original P value, and P < 0.05 was considered statistically significant.

### MELTF-AS1 regulated miR-1299

**Figure 5.**
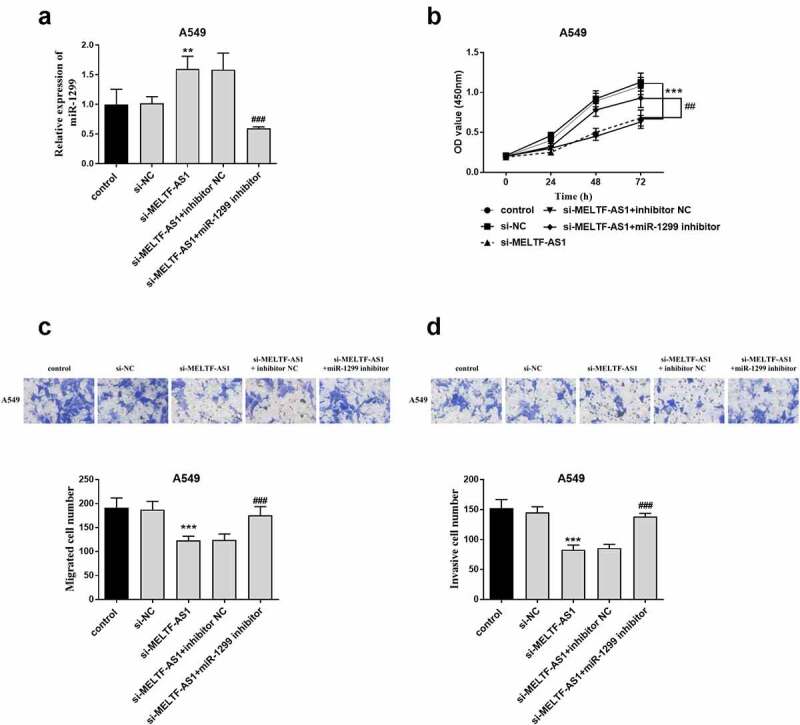
A549 cells were co-transfected with control, si-NC, si-MELTF-AS1, si-MELTF-AS1+ inhibitor NC or si-MELTF-AS1+ miR-1299 inhibitor. (a) si-MELTF-AS1+ miR-1299 inhibitor inhibited the relative expression level of miR-1299 by RT-qPCR. (b) CCK-8 detected A549 cell proliferation. (c) Transwell cell migration assay. (d) Cell infiltration was analyzed by Transwell. **P < 0.01, ***P < 0.001 vs si-NC; ^##^*P* < 0.01, ^###^*P* < 0.001 vs si-MELTF-AS1+ inhibitor NC.

To demonstrate that MELTF-AS1 affects the development of NSCLC by regulating the expression of miR-1299, A549 cells were co-transfected with control, si-NC, si-MELTF-AS1, si-MELTF-AS1+ inhibitor NC or si-MELTF-AS1+ miR-1299 inhibitor via Lipofectamine 2000. RT-qPCR results are shown in Figure 5(a), si-MELTF-AS1+ miR-1299 inhibitor inhibited the relative expression level of miR-1299. The proliferation of A549 cells was shown By the OD value curve in Figure 5(b), si-MELTF-AS1+ miR-1299 inhibitor reversed the inhibition of si-MELTF-AS1 on cell proliferation. Similarly, si-MELTF-AS1+ miR-1299 inhibitor promoted the level of cell proliferation and invasion (Figure 5(c,d)).

## Discussion

It is known that lung cancer incorporated small cell carcinoma (SCLC) and NSCLC, such as squamous cell carcinoma, adenocarcinoma and large cell carcinoma, which all belong to the latter [21,22]. Therefore, it is of great significance to explore the development of NSCLC and the further study of prognostic diagnosis.

As the molecular level is increasingly applied to the discovery and treatment of various cancers, researchers have shown that lncRNAs as prognostic marker may regulate and play a role in colorectal cancer [23], lung cancer [24], breast cancer [25], and bladder cancer [26], affecting the cancers progression and patient survival. In this study, lncRNA MELTF-AS1, an antisense RNA1 of melanin transferrin, is encoded by a gene located at the distal end of chromosome 3q, which is a chromosome amplified gene found in many cancers [27]. A study of seven lncRNAs with prognostic value found that risk signals including MELTF-AS1 can be used as prognostic indicators in patients with clear cell renal cell carcinoma (ccRCC), and may serve as potential prognostic biomarkers [28]. Besides, MELTF-AS1 has also been reported as a biomarker for the treatment of osteosarcoma and renal clear cell carcinoma [29], and is associated with the prognosis of NSCLC, so this study selected MELTF-AS1 as the research object for follow-up experiments.

This paper elaborated that MELTF-AS1 increased in NSCLC tissues and cells, which was also confirmed in the TCGA NSCLC cohort. It has been reported that some RNA transcripts, such as mRNA and lncRNA, competitively bind the same microRNA, communicated and regulated their expression levels through shared miRNAs. Thus, the upregulation mechanism of MELTF-AS1 expression in NSCLC was speculated, which was also explained by Ding et al. in the regulation of MELTF-AS1 on the treatment of osteosarcoma [29]. Kaplan-Meier analysis showed that low lncRNA MELTF-AS1 expression was more conducive to patient survival. Deng et al.’s research on NSCLC found that lncRNA AFAP1-AS1 expression in NSCLC tissues was markedly increased, and the survival time of patients with low AFAP1-AS1 expression would be longer than that of high [30]. Subsequently, the study of silencing MELTF-AS1 was carried out at the cellular level in vitro. Silencing MELTF-AS1 affected the proliferation ability, migration capability and invasion levels of SK-MES-1 and A549 cells, resulting in significant suppression of cell growth and NSCLC progression. Studies have shown that PITPNA-AS1 silencing also reduced the growth ability of A549 cells, suggesting that PITPNA-AS1 silencing has an inhibitory effect on NSCLC [31]. It should be noted that the lack of in vivo assay and in vivo targeted analysis of MELTF-AS1 were the limitations of this study. In addition, this study also suggested that lncRNA MELTF-AS1 affect the progression of NSCLC by targeting miR-1299. MiR-1299 has been shown to be an intervention suppressor gene for NSCLC treatment [32]. Moreover, it has also been confirmed that miR-1299 plays a corresponding role in ovarian cancer [33], prostate cancer [34] and other malignant tumors [35]. In this study, silencing MELTF-AS1 led to increased expression of miR-1299 in NSCLC cells. It was speculated that this was the result of MELTF-AS1 targeting miR-1299, and MELTF-AS1 inhibited miR-1299 expression level, thereby enhanced the process of NSCLC. In the investigation of Zhao et al., lncRNA RHPN1-AS1 promoted the growth and invasion of ovarian cancer by inhibiting miR-1299 and also showed the similarity of the relationship between lncRNA-miRNA [36]. Through the study of the regulation of miR-1299 by MELTF-AS1, combined with the report of Cao and colleagues, it was found that miR-1299 inhibits the progression of NSCLC through the EGFR/PI3K/Akt signaling pathway [32]. The above evidence implied that MELTF-AS1 has a negative regulatory effect on miR-1299, and MELTF-AS1 may serve as a biomarker of NSCLC and promote the progress of NSCLC.

## Conclusion

In summary, the current paper results indicated that lncRNA MELTF-AS1 contributed to the proliferation and growth of NSCLC cells by targeting miR-1299, which was helpful to the progress of NSCLC. As expected, MELTF-AS1 has the potential as a prognostic marker of NSCLC, which may provide a certain theoretical basis for the subsequent treatment of NSCLC.
